# Heme Oxygenase-1 Suppresses Wnt Signaling Pathway in Nonalcoholic Steatohepatitis-Related Liver Fibrosis

**DOI:** 10.1155/2020/4910601

**Published:** 2020-05-01

**Authors:** Jinghua Du, Weiguang Ren, Qingshan Zhang, Na Fu, Fang Han, Po Cui, Wencong Li, Lingbo Kong, Suxian Zhao, Rongqi Wang, Yuguo Zhang, Luting Yang, Li Kong, Yuemin Nan

**Affiliations:** Department of Traditional and Western Medical Hepatology, Third Hospital of Hebei Medical University, Shijiazhuang, China

## Abstract

**Methods:**

Mice were fed with a methionine-choline-deficient (MCD) diet for 8 weeks to induce steatohepatitis-related liver fibrosis and were treated with HO-1 inducer Hemin and inhibitor ZnPP. Mouse sera were collected for the biochemical analysis, and livers were obtained for further histological observation and gene expression analysis. HSC-T6 cells were cultured for the *in vitro* study and were administrated with Hemin and si-HO-1 to induce or inhibit the expression of HO-1. qPCR and Western blot were used to assess the mRNA and protein levels of genes.

**Results:**

MCD-fed mice developed marked macrovesicular steatosis, focal necrosis, and inflammatory infiltration and pericellular fibrosis in liver sections. Administration of Hemin could significantly ameliorate the severity of steatosis, inflammation, and fibrosis and also could decrease the serum ALT and AST. We demonstrated that HO-1 induction was able to downregulate the key regulator of the canonical Wnt pathway Wnt1 and the noncanonical Wnt pathway Wnt5a. The downstream factors of the Wnt pathway *β*-catenin and NFAT5 were inhibited by Hemin, but GSK-3*β* was upregulated compared to the MCD group, which were consistent with the *in vitro* study. Hemin markedly inhibited the TGF-*β*1/Smad signaling pathway in both *in vivo* and *in vitro* studies.

**Conclusion:**

Our study demonstrated that HO-1 inhibited the activation of canonical and noncanonical Wnt signaling pathways in NASH-related liver fibrosis. Thus, these results may suggest a new therapeutic strategy for NASH-related liver fibrosis.

## 1. Introduction

Nonalcoholic fatty liver disease (NAFLD) has been recognized as the most important cause of chronic liver disease in developed countries and has been of considerable interest in recent years [1]. NAFLD encompasses a histological spectrum ranging from simple steatosis and nonalcoholic steatohepatitis (NASH) to fibrosis and hepatocellular carcinoma [2]. Great progress has been made in this field, and the proposed second-hit theory is the generally accepted theory concerning the pathophysiology of NASH [3, 4]. Increased lipid peroxidation and reactive oxygen species (ROS) are two major contributors to the progression of NASH and related liver fibrosis [5–7]. Therefore, suppression of oxidative stress could provide a therapeutic alternative in the treatment of NASH-related liver fibrosis.

Heme oxygenase-1 (HO-1), known as heat shock protein 32, is one of the key antioxidant defenses that inhibit ROS by the degradation of heme to carbon monoxide (CO), biliverdin (BV), and iron [8]. Increasing HO-1 activity could reverse cellular oxidative stress and protect hepatocytes from ROS in liver damage [9, 10]. In the previous study, we have endeavored to investigate the relationship between HO-1 and NAFLD and demonstrated that the induction of HO-1 could eliminate steatohepatitis and hepatic fibrosis in the progression of NAFLD [11, 12], thus substantiating a significant role of HO-1 against heme-mediated steatohepatitis-related liver fibrosis. Nevertheless, even though the protective effects of HO-1 during NASH and NASH-related liver fibrosis have been established, the exact mechanism underlying between HO-1 and steatohepatitis-related liver fibrosis remains implicit.

The Wnt signaling pathway, which could be stimulated by hepatic stress, plays a pivotal role in the pathology and physiology of the liver [13]. It is revealed that Wnt signaling pathways are strongly related to hepatocellular processes, including proliferation, development, and carcinogenesis [14]. Emerging evidence implicates that Wnt signaling plays important roles in chronic liver disease, e.g., viral hepatitis, alcoholic liver disease, and NAFLD [15]. Moreover, the noncanonical signaling pathway contributes to the inflammation of NASH and plays a proinflammatory role in the process of NASH. In addition, serum Wnt5a levels correlate with the severity of hepatic steatosis and NASH [16]. Numerous studies demonstrate that Wnt signaling is critical for the fibrogenesis process in liver fibrosis [17, 18]. Recent studies have shown that ROS production could activate the Wnt/*β*-catenin signaling pathway [19]. However, the relation between the Wnt signaling pathway and oxidative stress remains unclear.

For this study, we have hypothesized that HO-1 could regulate the Wnt signaling pathway in dietary steatohepatitis-related liver fibrosis. To investigate this hypothesis, we established NASH animal models with a methionine-choline-deficient (MCD) diet and treated mice with HO-1 inducer Hemin and inhibitor ZnPP, simultaneously. The expression of Wnt and downstream genes was detected. We believe that understanding the interactions between the HO-1 and the Wnt signaling pathway in NASH-related fibrosis will lead to the development of therapeutic strategies to fight hepatic dysfunction.

## 2. Materials and Methods

### 2.1. Animals and Treatments

Male C57BL/6J mice were maintained in a 12 h light/dark cycle with free access to water and food *ad libitum* as previously described [20]. Mice were randomly divided into 4 groups (6 mice per group): (1) control group: mice were fed with diet supplemented with choline bitartrate (2 g/kg) and DL-methionine (3 g/kg) (Research Diets, Inc., NJ, New Brunswick, USA); (2) MCD group: mice were fed a methionine-choline-deficient (MCD) diet (Research Diets, Inc., NJ, New Brunswick, USA); (3) MCD+Hemin group: mice were fed a MCD diet and treated with HO-1 chemical inducer Hemin (30 *μ*mol/kg) by intraperitoneal (i.p.) injections three times per week; and (4) MCD+ZnPP group: mice were fed a MCD diet and treated with HO-1 inhibitor ZnPP-IX (20 *μ*mol/kg) by i.p. injections three times per week. At the end of the experiment for 8 weeks, all animals were fasted overnight before being euthanized. The livers were fixed in 10% formalin for histological analysis or snap-frozen in liquid nitrogen followed by storage at -80°C freezer for RNA and protein. All the protocols and procedures were carried out in accordance with the guidelines of the Hebei Committee for Care and Use of Laboratory Animals and were approved by the Animal Experimentation Ethics Committee of the Hebei Medical University.

### 2.2. Histological and Biochemical Analyses and ROS Assessment

Hematoxylin and eosin staining and Masson's Trichrome staining were performed on paraffin-embedded liver sections (5 *μ*m thick) to observe hepatic steatosis, inflammation, and fibrosis, respectively. The quantification of liver fibrosis was determined by METAVIR Scoring System. Serum ALT and AST levels were measured by the enzymatic kinetic method using an automatic biochemical analyzer (Olympus AU2700, Japan) according to the manufacturer's instructions. Hepatic ROS levels were assessed by flow cytometry by dihydrodichlorofluorescein diacetate (DCFH-DA, Nanjing Jiancheng Biotechnology Institute, China), the most sensitive probe that becomes fluorescent upon oxidation by ROS.

### 2.3. Cell Culture and Transient Transfection

The HSC-T6 cells were maintained in Dulbecco's modified Eagle's high glucose medium (DMEM), supplemented with 100 U/ml penicillin, 100 g/ml streptomycin (all from Life Technologies, Inc., Gaithersburg, MD), and 10% fetal bovine serum (Biological Industries, Kibbutz Beit Ha'Emek, Israel). The cells were incubated at 37°C in a humidified atmosphere with 5% CO_2_. Transient transfection was performed in 6-well plates. HSC-T6 cells were transfected with HO-1 siRNA (GenePharma, Shanghai) using a Lipofectamine 2000 transfection reagent (Invitrogen, Carlsbad, CA) according to the manufacturer's instructions. Four hours after treatment, the serum-free medium was replaced with complete medium for 24 h, and the cells were harvested.

### 2.4. Quantitative Real-Time Reverse Transcription Polymerase Chain Reaction (qRT-PCR)

Total RNA was isolated from frozen liver tissues and cell palate using a TRIzol Reagent (Invitrogen, Carlsbad, CA) according to the manufacturer's instructions. The first-strand cDNA was synthesized using a PrimeScript RT Master Mix (Takara Bio, RR036A). qRT-PCR were performed using the ABI 7500 sequence detection system (Applied Biosystems, Foster, CA) with a TB Green Premix Ex Taq II kit (Takara Bio, RR820A). The relative amount of each gene was measured using the 2^-*ΔΔ*Ct^ method, and gene relative expression was normalized to GAPDH. The specific primers were designed using Primer 5.0 and are shown in Table 1.

### 2.5. Western Blot

Total protein was extracted by RIPA (Solarbio, Beijing), and the concentration was measured by bicinchoninic acid (Solarbio). The nuclear and cytoplasmic fractions were isolated by a Nuclear Extraction Kit (Solarbio, Beijing, China) according to the manufacturer‘s instruction. 80 *μ*g/well proteins were loaded onto 10% SDS-PAGE for each sample, and proteins were transferred onto equilibrated polyvinylidene difluoride membranes (Millipore Corporation, Billerica, MA, USA) by electroblotting. Membranes were blocked with TBST containing 5% milk and incubated overnight at 4°C with primary antibodies against HO-1 (Bioworld Tech, LP, USA), Wnt1 (Abcam, Cambridge, MA, USA), Wnt5a (Novus Biologicals, Littleton, USA), *β*-catenin, GSK-3*β*, phospho-GSK-3*β* (Ser9) (ProteinTech Group, Chicago, USA), and NFAT5 (Santa Cruz, CA, USA). After incubation with the secondary antibody (ProteinTech), proteins expression was corrected by the amount of *β*-actin (ProteinTech) in the same sample. Lamin A (ProteinTech) and tubulin (ProteinTech) were used as the marker for nuclear and cytosolic proteins, respectively. The bands were quantified by scanning densitometry using Quantity One 4.6.3 software (Bio Rad).

### 2.6. Measurement of Apoptosis and Cell Proliferation

Apoptosis of HSC-T6 cells were assessed using an Annexin V-FITC/PI apoptosis detection kit (BD Bioscience) following the manufacturer's instruction by flow cytometry. To investigate the effect of HO-1 on the proliferation of HSC-T6 cells, five hours after treatment, HSC-T6 cells were reseeded on 96-well plates, at a density of 5 × 10^3^ cells per well for 1, 2, 3, 4, and 5 d. The cells were assessed for proliferation using the Cell Counting Kit-8 (CCK-8, Dojindo, Kumamoto, Japan), according to the manufacturer's instructions. The experiments were conducted three times, independently.

### 2.7. Statistical Analysis

All data are expressed as mean standard deviation (SD). Statistical analysis was carried out by one-way analysis of variance (ANOVA) and the Student-Newman-Keuls test for evaluating differences between groups using SPSS 19.0. *P* value of less than 0.05 was considered statistically significant.

## 3. Results

### 3.1. HO-1 Alleviated Hepatic Inflammation and Fibrosis in MCD Diet-Induced NASH-Related Liver Fibrosis

**Figure 1:**
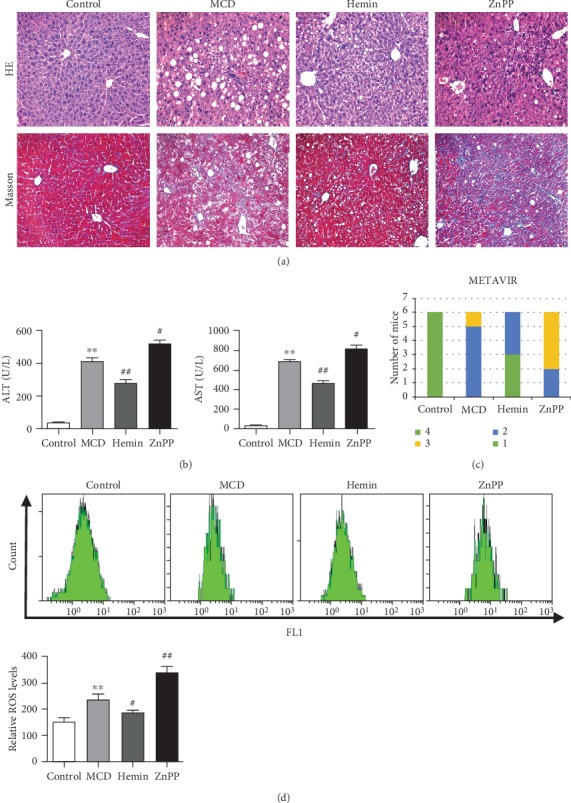
Mice fed with MCD diet developed steatohepatitis-related liver fibrosis. (a) Histological images of liver sections: H&E (top row, ×200) and Masson (bottom row, ×200). (b) Blood biochemical analysis of mice. (c) Quantification of liver fibrosis using the METAVIR Scoring System in each group. (d) The hepatic ROS levels of mice by flow cytometry. Values are mean ± SD, ^∗∗^*P* < 0.01 vs. the control group, ^#^*P* < 0.05, ^##^*P* < 0.001 vs. the MCD group.

Mice fed with a MCD diet for 8 weeks exhibited macrovesicular steatosis, ballooning degeneration, disordered lobule structure in Zone 3, focal necrosis, and inflammatory infiltration and pericellular fibrosis (Figure 1(a)). Administration of HO-1 inducer Hemin could significantly ameliorate the severity of steatosis, inflammation, and fibrosis (Figure 1(a)). Moreover, induction of HO-1 by Hemin markedly decreased the serum ALT and AST levels (Figure 1(b)), compared with the MCD diet-fed mice. In contrast, liver sections from mice treated with the HO-1 inhibitor ZnPP showed aggravated hepatic steatosis, inflammation, and fibrosis (Figure 1(a)) and further elevated serum transaminase levels (Figure 1(b)). These results demonstrated that HO-1 induction alleviated liver injury and hepatic fibrosis in MCD diet-induced steatohepatitis. To assess the degree of liver fibrosis, we quantified liver fibrosis with the METAVIR Scoring System. As shown in Figure 1(c), the MCD diet mice progress to more sever fibrosis than the control mice. Administrated Hemin could significantly decrease the liver fibrosis, and the HO-1 inhibitor could promote liver fibrosis as compared with the MCD diet-fed mice.

To further investigate the effect of HO-1 modulation on ROS in the pathogenesis of NASH, we determined hepatic ROS by flow cytometry in each group of mice. The results showed that MCD could significantly induce the increase of hepatic ROS. With the induction of HO-1 by Hemin, the ROS level was significantly decreased. And the hepatic ROS level of mice with the administration of ZnPP was further increased as compared with that of the MCD group mice (Figure 1(d)).

### 3.2. The Effects of HO-1 on Wnt Signaling Pathway in NASH-Related Liver Fibrosis Mice

**Figure 2:**
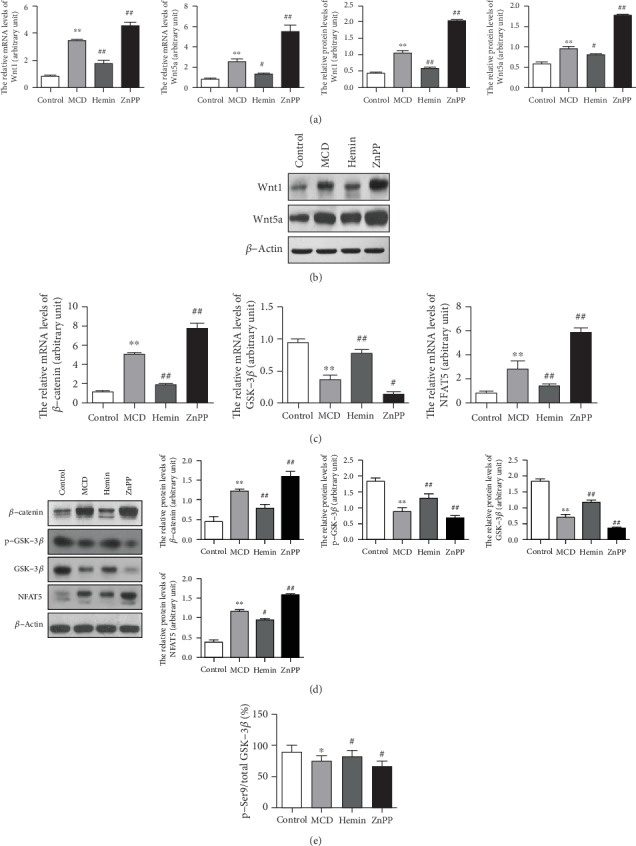
HO-1 inhibited Wnt signaling pathway and downstream effectors. (a) mRNA and (b) protein levels of Wnt1 and Wnt5a were detected by qPCR and Western blot, respectively. (c) mRNA and (d) protein levels of Wnt signaling pathway downstream effectors were detected by qPCR and Western blot, respectively. (e) Densitometry analysis shows the band density ratio of p-GSK-3*β* (Ser9) to total GSK-3*β*. Values are mean ± SD; ^∗^*P* < 0.05, ^∗∗^*P* < 0.01 vs. the control group, ^#^*P* < 0.05, ^##^*P* < 0.01 vs. the MCD group.

To address the role of HO-1 on Wnt signaling pathway, we investigated the hepatic expression of canonical and noncanonical Wnt pathways in MCD diet-induced steatohepatitis-related liver fibrosis. We demonstrated that HO-1 induction by Hemin was able to downregulate the key regulator of the canonical Wnt pathway Wnt1 and the noncanonical Wnt pathway Wnt5a (Figures 2(a) and 2(b)). As shown in Figures 2(c) and 2(d), *β*-catenin and NFAT5 were inhibited by the HO-1 inducer Hemin compared to those in the MCD group, but p-GSK-3*β* and GSK-3*β* expression was upregulated. However, the expression of Wnt1, Wnt5a, and downstream factors *β*-catenin and NFAT5 was further increased in mice treated with ZnPP, and the expression of p-GSK-3*β* and GSK-3*β* was decreased compared to that of the MCD group (Figure 2). Densitometry analysis shows the band density ratio of p-GSK-3*β* (Ser9) to total GSK-3*β* in Figure 2(e). These findings suggested that HO-1 could blunt the canonical and the noncanonical Wnt signaling pathway to prevent steatohepatitis-related liver fibrosis.

### 3.3. HO-1 Suppressed TGF-*β* Signaling Pathway in NASH-Related Liver Fibrosis Mice

**Figure 3:**
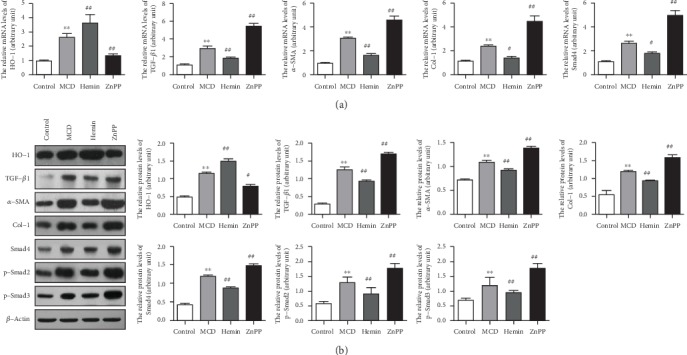
HO-1 suppressed the TGF-*β* signaling pathway in NASH-related liver fibrosis mice. (a) Hepatic mRNA and (b) protein levels of fibrosis-related genes TGF-*β*1, *α*-SMA, Col-1, Smad4, p-Smad2, and p-Smad3. *β*-Actin was used as the loading control. Values are mean ± SD; ^∗∗^*P* < 0.01 vs. the control group; ^#^*P* < 0.05, ^##^*P* < 0.01 vs. the MCD group.

To investigate the mechanisms of HO-1 on steatohepatitis-related liver fibrosis, we assessed the hepatic expression levels of fibrogenic factors. Hepatic mRNA (Figure 3(a)) and protein (Figure 3(b)) levels of transforming growth factor-*β*1 (TGF-*β*1), alpha-smooth muscle actin (*α*-SMA), collagen I (Col-1), Smad4, phosphorylated Smad2 (p-Smad2), and phosphorylated Smad3 (p-Smad3) were significantly increased in MCD diet-fed mice, which were blunted by Hemin administration. Conversely, hepatic expressions of TGF-*β*1, *α*-SMA, Col-1, and Smad4 were further increased by the treatment with ZnPP as compared with those of MCD-induced steatohepatitis-related liver fibrosis (Figures 3(a) and 3(b)).

### 3.4. Induction of HO-1 by Hemin Suppressed Wnt Signaling Pathway in HSC-T6 Cells

**Figure 4:**
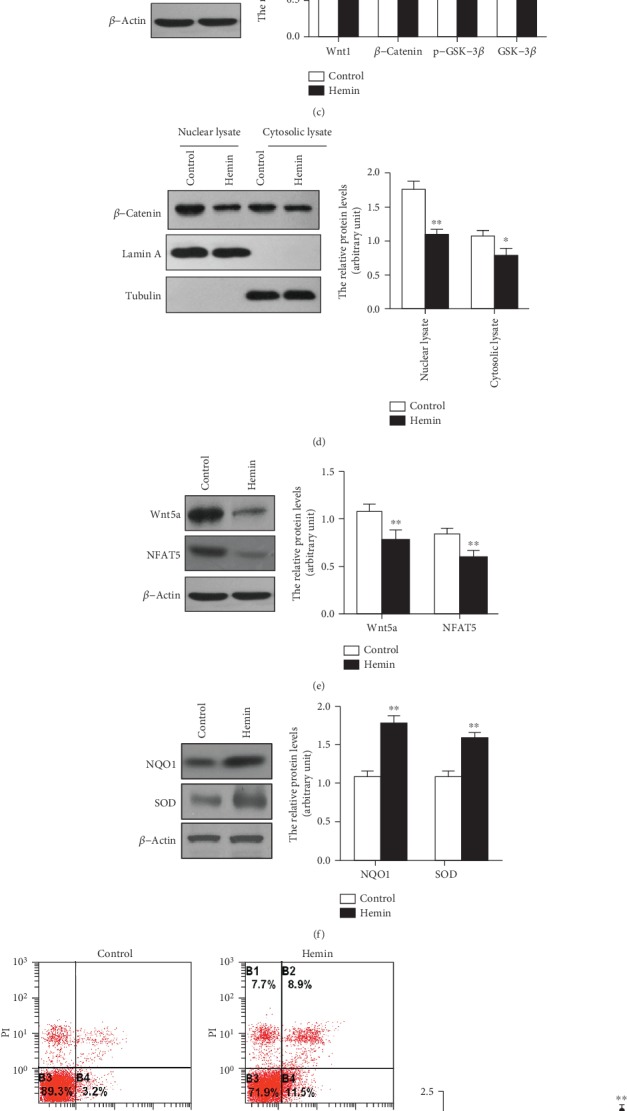
Induction of HO-1 expression suppressed the Wnt signaling pathway in HSC-T6. (a) Expression levels of HO-1 after Hemin administration at 0, 6, 12, and 24 hours in HSC-T6. *β*-Actin was used as the loading control. Values are mean ± SD; ^∗∗^*P* < 0.01 vs. 0 h. (b) Then, we administrated HSC-T6 with 0, 20, 40, and 60 *μ*M Hemin for 12 hours. With the increased concentration of Hemin, the HO-1 level was not further increased in HSC-T6 at the concentration of 40 *μ*M. *β*-Actin was used as the loading control. Values are mean ± SD; ^∗∗^*P* < 0.01 vs. 0 *μ*m. (c) The HSC-T6 were treated with 40 *μ*M Hemin for 12 hours, and the expression of Wnt1 and downstream effectors was determined by Western blot. (d) The nuclear and cytoplasmic fractions were isolated to detect the expression of *β*-catenin. Lamin A and tubulin were used as the marker for nuclear and cytosolic proteins, respectively. (e) The expression of Wnt5a and NFAT5 were determined by Western blot. (f) The protein levels of oxidative stress markers NQO1 and SOD. (g) The flow cytometry data of Annexin V-FITC/PI to assessing apoptosis of HSC-T6 after the administration of Hemin. (h) Hemin inhibited HSC-T6 cell growth as determined by the CCK-8 assay. (i) The protein levels of fibrosis-related genes were performed by Western blot. *β*-Actin was used as the loading control. Values are mean ± SD; ^∗^*P* < 0.05, ^∗∗^*P* < 0.01 vs. the control group.

To determine the effects of HO-1 induction on the Wnt pathway in HSC-T6, we treated HSC-T6 with the HO-1 inducer Hemin and harvested cells at 0, 6, 12, and 24 hours after cell treatment. HO-1 mRNA and protein levels began to increase by 6 hours, and a more striking increase in HO-1 was evident after 12 hours of Hemin treatment, which was not further increased after 24 hours of treatment (Figure 4(a)). We then administrated HSC-T6 with 0, 20, 40, and 60 *μ*M Hemin for 12 hours. We found that with the increased concentration of Hemin, the HO-1 level was not further increased in HSC-T6 at the concentration of 40 *μ*M (Figure 4(b)). Thus, HSC-T6 were treated with 40 *μ*M Hemin for 12 hours in the following study. After Hemin treatment, the protein expressions of Wnt1 and the downstream factor *β*-catenin were significantly downregulated. To examine the effects of HO-1 on *β*-catenin accumulation and GSK-3*β* activation, we quantified the cytosolic and nuclear *β*-catenin and total and p-GSK-3*β* at Ser9 by Western blot. The results showed that nuclear and cytosolic *β*-catenin were reduced after Hemin administration. More importantly, there was a more significant decrease in nuclear *β*-catenin (Figure 4(d)). However, p-GSK-3*β* and GSK-3*β* expressions were upregulated in Hemin-treated HSC-T6 compared to those of the control (Figure 4(c) and Supplementary Figure 1A). These data suggested that Hemin could inhibit the accumulation and nuclear translocation of *β*-catenin. As shown in Figure 4(e), the expressions of Wnt5a and NFAT5 were significantly decreased in Hemin-treated HSC-T6 cells.

As shown in Figure 4(f), with the administration of Hemin, the oxidative stress marker NAD(P)H Dehydrogenase, Quinone 1 (NQO1) and superoxide dismutase (SOD) were significantly increased, suggesting that the induction of HO-1 could decrease the ROS level in HSC-T6 cells. To further study the effect of HO-1 on apoptosis and cell growth of HSCs, we treated HSC-T6 with Hemin, determined the apoptosis of cells with Annexin V-FITC/PI by flow cytometry, and investigated cell growth by the CCK-8 assay. The results showed that Hemin could promote apoptosis (Figure 4(g)) and inhibit the cell growth of HSC-T6 (Figure 4(h)). In addition, we found that Hemin markedly decreased the levels of TGF-*β*1, *α*-SMA, Col-1, Smad4, p-Smad2, and p-Smad3 in HSC-T6 (Figure 4(i)). These data suggested that induction of HO-1 by Hemin could suppress the function of HSC via inhibiting the TGF-*β*/Smad signaling pathway by blocking canonical and noncanonical Wnt signaling pathways.

### 3.5. Knockdown HO-1 Stimulated Wnt Signaling Pathway in HSC-T6 Cells

**Figure 5:**
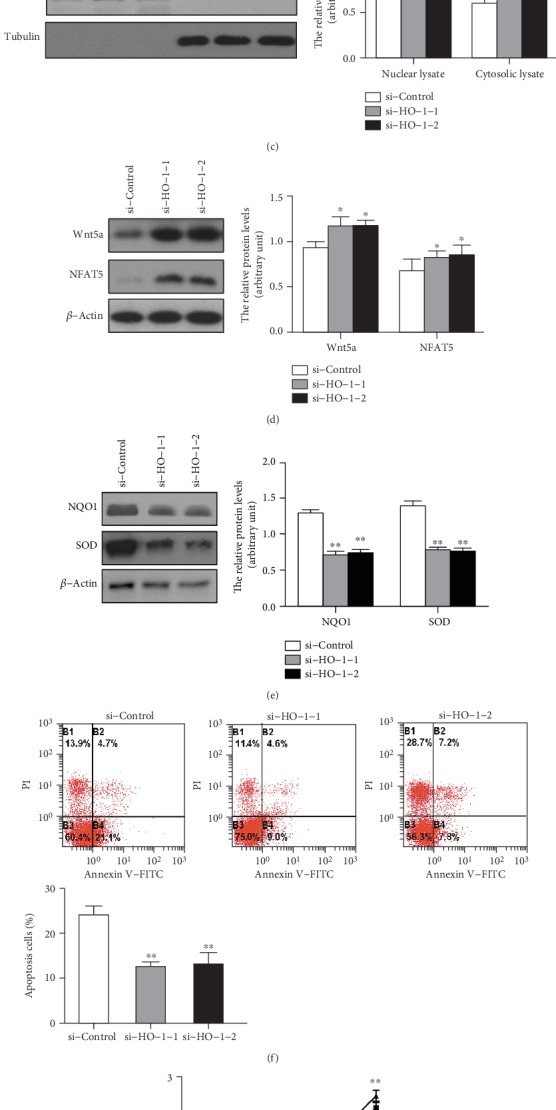
Knockdown HO-1 stimulated the Wnt signaling pathway in HSC-T6 cells. We transfected si-HO-1 into HSC-T6 to knockdown HO-1 expression. (a) Expression of HO-1 after transfected with HO-1 siRNA. (b) Wnt1 and downstream factor protein levels after being transfected with si-HO-1. (c) The nuclear and cytoplasmic fractions were isolated to detect the expression of *β*-catenin. Lamin A and tubulin were used as the marker for nuclear and cytosolic proteins, respectively. (d) Wnt5a and downstream factor protein levels after being transfected with si-HO-1. (e) The protein levels of oxidative stress markers NQO1 and SOD. (f) Inhibition of HO-1 decreased the apoptosis levels as determined by flow cytometry of Annexin V-FITC/PI. (g) Inhibition HO-1 expression by si-HO-1 promoted cell growth of HSC-T6. (h) The protein levels of fibrosis-related genes were performed by Western blot. *β*-Actin was used as the loading control. Values are mean ± SD; ^∗^*P* < 0.05, ^∗∗^*P* < 0.01 vs. the si-Control group.

We next investigated the effect of HO-1 knockdown on the Wnt signaling pathway in HSC-T6. We found that by transfecting HSCs with si-HO-1, the protein level of HO-1 was significantly decreased compared to that of the control (Figure 5(a)). After transfecting HSC-T6 with si-HO-1, the expressions of Wnt1 and Wnt5a were increased compared to those of the control (Figures 5(b) and 5(d)). The downstream factors *β*-catenin and NFAT5 were significantly upregulated, but p-GSK-3*β* and GSK-3*β* expressions were downregulated in HSC-T6 after si-HO-1 transfection compared to those of the control (Figure 5(b) and supplementary Figure 1B). We further investigated the nuclear and cytosolic *β*-catenin expression and found that the knockdown of HO-1 could significantly increase *β*-catenin translocation to the nucleus (Figure 5(c)). Furthermore, si-HO-1 could significantly decrease the ROS level of HSC-T6 by decreasing the oxidative stress markers NQO1 and SOD (Figure 5(e)) and inhibit the apoptosis of HSC as determined by flow cytometry of Annexin V-FITC/PI (Figure 5(f)). We also found that si-HO-1 significantly increased the cell growth of HSC-T6 cells compared to si-Control (Figure 5(g)). Additionally, the expressions of TGF-*β*1, *α*-SMA, Col-1, Smad4, p-Smad2, and p-Smad3 were increased in si-HO-1-transfected HSC-T6 (Figure 5(h)). These results demonstrate that knocking down HO-1 could stimulate the Wnt signaling pathway to induce fibrogenic activity in HSC-T6 cells.

## 4. Discussion

To clarify the pathophysiological mechanisms of NASH, an ideal NASH animal model should replicate the pathophysiology of human patients, including insulin resistance, steatohepatitis, and liver fibrosis. The MCD diet is one of the widely employed dietary models for NAFLD [21]. The MCD diet could better mimic the pathological features of severe human NASH, like steatohepatitis and NASH-related liver fibrosis, than other dietary animal models [22, 23]. In the present study, we fed C57BL/6J mice with MCD for 8 weeks to induce steatohepatitis-related liver fibrosis. Serum ALT and AST levels were significantly increased, and histological evidence of steatohepatitis, inflammation, and perisinusoidal fibrosis has been observed in the liver sections of MCD diet-fed mice, which were consistent with our previous studies [24]. The MCD dietary model has the main advantage of effectively replicating severe steatohepatitis and liver fibrosis in a shorter time than other NASH animal models [25].

In the progression of NAFLD, continuous oxidative stress leads to the imbalance of ROS and the antioxidant capacity of hepatocytes and then causes lipid peroxidation, intracellular organelle damage, and activation of HSCs [6]. Our previous studies have shown that HO-1 plays a very important role in NASH-related liver fibrosis, and it may act as an antioxidative mediator under stress conditions in NASH [12]. In injured hepatocytes, induced HO-1 may lead to adaptive stress reaction and may be the response to increased liver oxidative stress, protecting hepatocytes from oxidative injury [26]. It has been suggested that Hemin and ZnPP regulate heme catabolism through the induction and inhibition of HO-1 and thus regulate liver damage and fibrogenesis [27]. In our study, HO-1 was upregulated in the MCD diet-fed mice, and higher ATL and AST levels were detected in this group. However, in the mice treated with Hemin, ALT and AST levels were significantly decreased with the induction of HO-1 as compared with the MCD group. After being treated with ZnPP, ALT and AST levels were increased, suggesting more aggravated liver injury as compared with the MCD group, consistent with our previous study [11, 12]. This remarkable finding prompted further investigation into targets that link HO-1 with steatohepatitis-related liver fibrosis.

Wnts belong to a family of secreted proteins involved in cell proliferation, differentiation, and activity [28]. Recently, the novel role of the Wnt signaling pathway in the pathogenesis of NASH has been given much more attention. The Wnt signaling pathway can be subclassified into the canonical or Wnt/*β*-catenin pathway and the noncanonical Wnt pathway [29]. However, recent studies highlight a pivotal role of the canonical Wnt signaling pathway in liver fibrogenesis [15, 30, 31]. Accordingly, the major role of Wnt signaling is to inhibit the differentiation of adipocytes and maintain preadipocytes in an undifferentiated state in steatohepatitis [32]. However, the role of Wnt signaling in steatohepatitis-related liver fibrosis remains unclear. Thus, upregulated Wnt1 and Wnt5a in MCD diet-induced steatofibrosis mice may account for the steatohepatitis-related liver fibrosis in the present study. Activated Wnt1 and Wnt5a then stimulated the canonical and noncanonical Wnt signaling pathway downstream factors *β*-catenin, GSK-3*β*, and NFAT5, which was evident by the *β*-catenin, NFAT5 and inhibition of the phosphorylation of GSK-3*β* in MCD diet-fed mice. In the canonical Wnt signaling pathway, activated Wnt1 binds to its receptor frizzled and then is stimulated by GSK-3*β* phosphorylation, preventing the major downstream effector *β*-catenin phosphorylation, which subsequently translocates to the nucleus from the cytoplasm [33]. Accumulated evidence showed that canonical Wnt signaling was activated in HSCs and their downstream elements of Wnt signaling were upregulated in fibrogenesis [34]. Accumulated evidence showed that mice with conditional knockout of *β*-catenin in hepatocytes developed more severe steatohepatitis and fibrosis with MCD diet compared with wild-type mice [35]. Early research also revealed that the noncanonical Wnt pathway was activated in steatohepatitis in high-cholesterol diet-induced NASH mice [36]. Researchers used microarrays to investigate differentially expressed genes between activated HSCs and quiescent HSCs and have found that Wnt5a was significantly upregulated [37]. Consistent with these, another study has identified that Wnt5a was increased in activated HSCs rather than in the quiescent HSCs [38]. We monitored TGF-*β* expression, which is a key factor in activating quiescent HSCs. TGF-*β* has been implicated to be activated by the Wnt signaling pathway [39]. Paralleled with the altered Wnt pathway, induction of TGF-*β*, *α*-SMA, Col-1, Smad4, p-Smad2, and p-Smad3 was detected in in the MCD diet-induced steatofibrosis. Thus, as the causal link between Wnt signaling and liver fibrosis, inhibition of the Wnt signaling pathway may be potential therapeutic targets for preventing steatohepatitis-related liver fibrosis.

As mentioned above, diet-induced steatohepatitis could upregulate HO-1 and the Wnt signaling pathway. However, the effects of HO-1 modulation on the Wnt signaling pathway have seldom been referred to in NAFLD. In the present study, the association between HO-1 and the Wnt signaling pathway in steatohepatitis-related liver fibrosis is a novel finding for further exploring the pathogenesis of NASH. Our investigations demonstrated that targeted induction of HO-1 by Hemin could suppress the Wnt signaling pathway, including the canonical Wnt1 pathway and the noncanonical Wnt5a pathway both *in vivo* and *in vitro*. Impaired Wnt1 and Wnt5a then regulate their downstream targets *β*-catenin, GSK-3*β*, and NFAT5 expression. These results were supported by a recent study that demonstrated that overexpression of HO-1 suppressed the activation of canonical Wnt/*β*-catenin signaling in renal fibrosis [40]. Other researches illustrated that overexpression of the HO-1/Wnt canonical signaling pathway could enhance neuroprotection and demonstrated a link between HO-1 and the canonical Wnt signaling pathway [41]. Strikingly, the current study was the first report that elucidated the connection of HO-1 and Wnt signaling in NASH-related liver fibrosis. Moreover, we also investigated the effect of HO-1 targeted modulation on cell apoptosis and cell growth. With the induction of the HO-1 expression, cell apoptosis was promoted and cell proliferation was inhibited. These data approved the protective role of HO-1 on the pathogenesis of NASH-related fibrosis by suppressing cell growth and enhancing cell apoptosis of HSCs. Meanwhile, in the current study, fibrogenic genes, such as TGF-*β*, *α*-SMA, Col-1, Smad4, p-Smad2, and p-Smad3, were significantly inhibited by the HO-1 induction in HSC-T6 with the treatment of Hemin, and transfection of si-HO-1 could further upregulate the expression of these fibrogenic genes, which were consistent with the *in vivo* study. These results demonstrated that HO-1 may play an antifibrotic role partially via suppressing the Wnt signaling pathway in steatohepatitis-related liver fibrosis.

In conclusion, our study demonstrated that the Wnt signaling pathway plays a pivotal role in the pathogenesis of diet-induced steatohepatitis-related liver fibrosis. Moreover, it is evident for the first time that HO-1 inhibited the activation of the canonical and noncanonical Wnt signaling pathways in NASH-related liver fibrosis. Thus, these findings may suggest a new therapeutic strategy for NASH-related liver fibrosis.

## Figures and Tables

**Table 1:** Primers for quantitative real-time PCR analysis.

Gene	Length of production (bp)	Primers
Wnt1	81	F 5′-CTCTTTGGCCGAGAGTTCGT-3′
		R 5′-TGCCTCGTTGTTGTGAAGGT-3′
Wnt5a	80	F 5′-GTTGCTCCGGCCCAGAAG-3′
		R 5′-TTGGAAGACATGGCACCTCC-3′
*β*-Catenin	125	F 5′-GTCAGTGCAGGAGGCCG-3′
		R 5′-GGCCATGTCCAACTCCATCA-3′
GSK-3*β*	146	F 5′-AGCTCTGATTGGCCACTGTC-3′
		R 5′-TAACCAATGAGGAGACGCCG-3′
NFAT5	121	F 5′-GCAGCCTCCAATCTCACACA-3′
		R 5′-GCAAGCGTCTGGGGTTATGT-3′
HO-1	82	F 5′-AAGCTTTTGGGGTCCCTAGC-3′
		R 5′-GGTGAGGGAACTGTGTCAGG-3′
TGF-*β*1	111	F 5′-CATTGCAGTGGTTTGGGGTG-3′
		R 5′-TCTGGAAAGGACCGTTGTCG-3′
*α*-SMA	156	F 5′-GTACCACCATGTACCCAGGC-3′
		R 5′-GCTGGAAGGTAGACAGCGAA-3′
Col-1	198	F 5′-TTCTCCTGGCAAAGACGGAC-3′
		R 5′-CGGCCACCATCTTGAGACTT-3′
Smad4	70	F 5′-CTTCCGACAAGTTGGCAGC-3′
		R 5′-CCCGAGCCGCTCCGTTA-3′
GAPDH	112	F 5′-CAAGAAGGTGGTGAAGCAGG-3′
		R 5′-AAAGGTGGAGGAGTGGGTGT-3′

## Data Availability

The data used to support the findings of this study are available from the corresponding author upon request.

## Supplementary Materials

Supplementary Materials Supplementary Figure 1: densitometry analysis of the band density ratio of p-Ser9/total GSK-3*β*. (A) The HSC-T6 were treated with 40 *μ*M Hemin for 12 hours. The band density ratio of p-Ser9/total GSK-3*β* was determined. Values are mean ± SD; ^∗^*P* < 0.05 vs. the control group. (B) We transfected si-HO-1 into HSC-T6 to knock down the HO-1 expression. The band density ratio of p-Ser9/total GSK-3*β* was determined. Values are mean ± SD; ^∗^*P* < 0.05 vs. the si-Control group.

## References

[1] Byrne C. D., Targher G. (2015). NAFLD: a multisystem disease. *Journal of Hepatology*.

[2] Cobbina E., Akhlaghi F. (2017). Non-alcoholic fatty liver disease (NAFLD) - pathogenesis, classification, and effect on drug metabolizing enzymes and transporters. *Drug Metabolism Reviews*.

[3] Berlanga A., Guiu-Jurado E., Porras J. A., Auguet T. (2014). Molecular pathways in non-alcoholic fatty liver disease. *Clinical and Experimental Gastroenterology*.

[4] Lake A. D., Novak P., Hardwick R. N. (2014). The adaptive endoplasmic reticulum stress response to lipotoxicity in progressive human nonalcoholic fatty liver disease. *Toxicological Sciences*.

[5] Bellanti F., Villani R., Facciorusso A., Vendemiale G., Serviddio G. (2017). Lipid oxidation products in the pathogenesis of non-alcoholic steatohepatitis. *Free Radical Biology & Medicine*.

[6] Sumida Y., Niki E., Naito Y., Yoshikawa T. (2013). Involvement of free radicals and oxidative stress in NAFLD/NASH. *Free Radical Research*.

[7] Paradies G., Paradies V., Ruggiero F. M., Petrosillo G. (2014). Oxidative stress, cardiolipin and mitochondrial dysfunction in nonalcoholic fatty liver disease. *World Journal of Gastroenterology*.

[8] Vanella L., Barbagallo I., Tibullo D., Forte S., Zappalà A., Volti G. L. (2016). The non-canonical functions of the heme oxygenases. *Oncotarget*.

[9] Yu M., Wang D., Xu M. (2014). Quinocetone-induced Nrf2/HO-1 pathway suppression aggravates hepatocyte damage of Sprague-Dawley rats. *Food and Chemical Toxicology*.

[10] Paudel P., Jung H. A., Choi J. S. (2018). Anthraquinone and naphthopyrone glycosides from Cassia obtusifolia seeds mediate hepatoprotection via Nrf2-mediated HO-1 activation and MAPK modulation. *Archives of Pharmacal Research*.

[11] Nan Y., Wang R., Zhao S. (2010). Heme oxygenase-1 prevents non-alcoholic steatohepatitis through suppressing hepatocyte apoptosis in mice. *Lipids in Health and Disease*.

[12] Wang R. Q., Nan Y. M., Wu W. J. (2011). Induction of heme oxygenase-1 protects against nutritional fibrosing steatohepatitis in mice. *Lipids in Health and Disease*.

[13] Ma Z. G., Lv X. D., Zhan L. L. (2016). Human urokinase-type plasminogen activator gene-modified bone marrow-derived mesenchymal stem cells attenuate liver fibrosis in rats by down-regulating the Wnt signaling pathway. *World Journal of Gastroenterology*.

[14] Monga S. P. (2015). *β*-Catenin Signaling and Roles in Liver Homeostasis, Injury, and Tumorigenesis. *Gastroenterology*.

[15] Wang J. N., Li L., Li L. Y., Yan Q., Li J., Xu T. (2018). Emerging role and therapeutic implication of Wnt signaling pathways in liver fibrosis. *Gene*.

[16] Ackers I., Malgor R. (2018). Interrelationship of canonical and non-canonical Wnt signalling pathways in chronic metabolic diseases. *Diabetes & Vascular Disease Research*.

[17] Zhang F., Lu C., Xu W. (2016). Curcumin raises lipid content by Wnt pathway in hepatic stellate cell. *The Journal of Surgical Research*.

[18] Arellanes-Robledo J., Reyes-Gordillo K., Shah R. (2013). Fibrogenic actions of acetaldehyde are *β*-catenin dependent but wingless independent: a critical role of nucleoredoxin and reactive oxygen species in human hepatic stellate cells. *Free Radical Biology & Medicine*.

[19] Zhang D. Y., Pan Y., Zhang C. (2013). Wnt/*β*-catenin signaling induces the aging of mesenchymal stem cells through promoting the ROS production. *Molecular and Cellular Biochemistry*.

[20] Jia Y. H., Wang R. Q., Mi H. M. (2012). Fuzheng Huayu recipe prevents nutritional fibrosing steatohepatitis in mice. *Lipids in Health and Disease*.

[21] Ibrahim S. H., Hirsova P., Malhi H., Gores G. J. (2016). Animal models of nonalcoholic steatohepatitis: eat, delete, and inflame. *Digestive Diseases and Sciences*.

[22] Lau J. K., Zhang X., Yu J. (2017). Animal models of non-alcoholic fatty liver disease: current perspectives and recent advances. *The Journal of Pathology*.

[23] Itagaki H., Shimizu K., Morikawa S., Ogawa K., Ezaki T. (2013). Morphological and functional characterization of nonalcoholic fatty liver disease induced by a methionine-choline-deficient diet in c57bl/6 mice. *International Journal of Clinical and Experimental Pathology*.

[24] Nan Y. M., Han F., Kong L. B. (2011). Adenovirus-mediated peroxisome proliferator activated receptor gamma overexpression prevents nutritional fibrotic steatohepatitis in mice. *Scandinavian Journal of Gastroenterology*.

[25] Sanches S. C. L., Ramalho L. N. Z., Augusto M. J., da Silva D. M., Ramalho F. S. (2015). Nonalcoholic steatohepatitis: a search for factual animal models. *BioMed Research International*.

[26] Sodhi K., Puri N., Favero G. (2015). Fructose mediated non-alcoholic fatty liver is attenuated by HO-1-SIRT1 module in murine hepatocytes and mice fed a high fructose diet. *PLoS One*.

[27] Kim C. S., Kwon Y., Choe S. Y. (2015). Quercetin reduces obesity-induced hepatosteatosis by enhancing mitochondrial oxidative metabolism via heme oxygenase-1. *Nutrition & Metabolism*.

[28] Zhan T., Rindtorff N., Boutros M. (2017). Wnt signaling in cancer. *Oncogene*.

[29] Clevers H., Nusse R. (2012). Wnt/*β*-catenin signaling and disease. *Cell*.

[30] Miao C. G., Yang Y. Y., He X. (2013). Wnt signaling in liver fibrosis: progress, challenges and potential directions. *Biochimie*.

[31] Beljaars L., Daliri S., Dijkhuizen C., Poelstra K., Gosens R. (2017). WNT-5A regulates TGF-*β*-related activities in liver fibrosis. *American Journal of Physiology. Gastrointestinal and Liver Physiology*.

[32] Christodoulides C., Lagathu C., Sethi J. K., Vidal-Puig A. (2009). Adipogenesis and WNT signalling. *Trends in Endocrinology and Metabolism*.

[33] Nusse R., Clevers H. (2017). Wnt/*β*-catenin signaling, disease, and emerging therapeutic modalities. *Cell*.

[34] Corbett L., Mann J., Mann D. A. (2015). Non-canonical Wnt predominates in activated rat hepatic stellate cells, influencing HSC survival and paracrine stimulation of Kupffer cells. *PLoS One*.

[35] Thompson M. D., Monga S. P. (2007). WNT/beta-catenin signaling in liver health and disease. *Hepatology*.

[36] Wang S., Song K., Srivastava R. (2015). Nonalcoholic fatty liver disease induced by noncanonical Wnt and its rescue by Wnt3a. *The FASEB Journal*.

[37] Jiang F., Parsons C. J., Stefanovic B. (2006). Gene expression profile of quiescent and activated rat hepatic stellate cells implicates Wnt signaling pathway in activation. *Journal of Hepatology*.

[38] Cheng J. H., She H., Han Y. P. (2008). Wnt antagonism inhibits hepatic stellate cell activation and liver fibrosis. *American Journal of Physiology. Gastrointestinal and Liver Physiology*.

[39] Kumawat K., Menzen M. H., Bos I. S. (2013). Noncanonical WNT-5A signaling regulates TGF-*β*-induced extracellular matrix production by airway smooth muscle cells. *The FASEB Journal*.

[40] Chen X., Wei S. Y., Li J. S. (2016). Overexpression of heme oxygenase-1 prevents renal interstitial inflammation and fibrosis induced by unilateral ureter obstruction. *PLoS One*.

[41] Tulsulkar J., Ward A., Shah Z. A. (2017). HO1 and Wnt expression is independently regulated in female mice brains following permanent ischemic brain injury. *Brain Research*.

