# Supplementary figures and images for: Heme Oxygenase-1 Suppresses Wnt Signaling Pathway in Nonalcoholic Steatohepatitis-Related Liver Fibrosis
Source: Biomed Res Int. 2020 May 1;2020:4910601. doi: 10.1155/2020/4910601 (PMC7212281; doi:10.1155/2020/4910601)

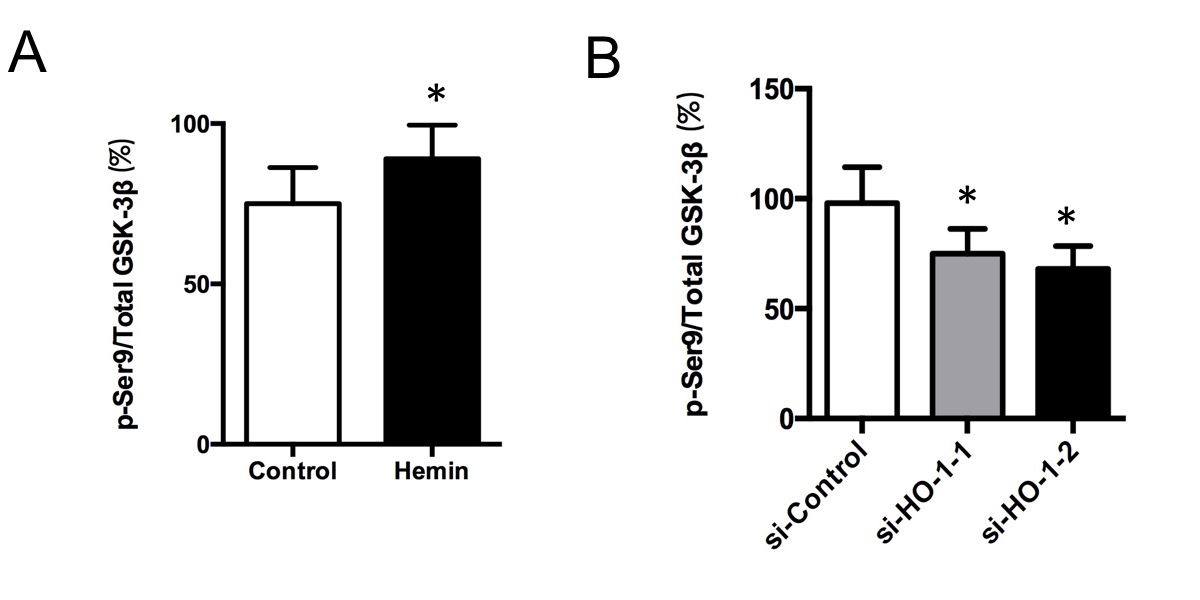

Supplement: Supplementary Materials — Supplementary Figure 1: densitometry analysis of the band density ratio of p-Ser9/total GSK-3β. (A) The HSC-T6 were treated with 40 μM Hemin for 12 hours. The band density ratio of p-Ser9/total GSK-3β was determined. Values are mean ± SD; ∗P < 0.05 vs. the control group. (B) We transfected si-HO-1 into HSC-T6 to knock down the HO-1 expression. The band density ratio of p-Ser9/total GSK-3β was determined. Values are mean ± SD; ∗P < 0.05 vs. the si-Control group. [file 4910601.f1.jpg]
